# The Spectrum of *PAX6* Mutations and Genotype-Phenotype Correlations in the Eye

**DOI:** 10.3390/genes10121050

**Published:** 2019-12-17

**Authors:** Dulce Lima Cunha, Gavin Arno, Marta Corton, Mariya Moosajee

**Affiliations:** 1 Institute of Ophthalmology, UCL, London EC1V 9EL, UK; 2 Moorfields Eye Hospital NHS Foundation Trust, London EC1V 2PD, UK; 3 Great Ormond Street Hospital for Children NHS Foundation Trust, London WC1N 3JH, UK; 4 Department of Genetics & Genomics, Instituto de Investigación Sanitaria-Fundación Jiménez Díaz University Hospital—Universidad Autónoma de Madrid (IIS-FJD, UAM), 28040 Madrid, Spain; 5 Centre for Biomedical Network Research on Rare Diseases (CIBERER), 28029 Madrid, Spain

**Keywords:** aniridia, enhancers, genotype-phenotype correlations, haploinsufficiency, *PAX6*, paired domain, microphthalmia, non-coding variants, premature termination codon, regulatory regions

## Abstract

The transcription factor PAX6 is essential in ocular development in vertebrates, being considered the master regulator of the eye. During eye development, it is essential for the correct patterning and formation of the multi-layered optic cup and it is involved in the developing lens and corneal epithelium. In adulthood, it is mostly expressed in cornea, iris, and lens. *PAX6* is a dosage-sensitive gene and it is highly regulated by several elements located upstream, downstream, and within the gene. There are more than 500 different mutations described to affect *PAX6* and its regulatory regions, the majority of which lead to *PAX6* haploinsufficiency, causing several ocular and systemic abnormalities. Aniridia is an autosomal dominant disorder that is marked by the complete or partial absence of the iris, foveal hypoplasia, and nystagmus, and is caused by heterozygous *PAX6* mutations. Other ocular abnormalities have also been associated with *PAX6* changes, and genotype-phenotype correlations are emerging. This review will cover recent advancements in *PAX6* regulation, particularly the role of several enhancers that are known to regulate *PAX6* during eye development and disease. We will also present an updated overview of the mutation spectrum, where an increasing number of mutations in the non-coding regions have been reported. Novel genotype-phenotype correlations will also be discussed.

## 1. Introduction

The human paired box 6, as coded by the *PAX6* gene, is a member of the PAX family of transcription factors, which are evolutionarily highly conserved among metazoans and are characterized by the presence of a conserved DNA-binding domain, the paired domain [1]. Positional cloning identified it in the early 1990s [2]. At the same time, murine *Pax6* was also identified by the screening of mouse embryonic expression libraries [3] and associated as the causal gene of an heterozygous *Sey* mouse strain (*Sey*^+/−^) [4], which presented with microphthalmia, iris hypoplasia, cataracts, and corneal opacifications, resembling human developmental eye disorder aniridia [5]. Further *PAX6* homologous genes were later identified in zebrafish (*pax6a* and *pax6b*) [6], quail [7], and *Drosophila* (*ey, toy, eyg,* and *toe*) [8]. 

PAX6 is considered to be the master regulator of the eye, since the overexpression of the gene resulted in ectopic eye formation in both *Drosophila* and *Xenopus* [9,10]. During early eye development, *PAX6* is expressed on the surface and neural ectoderm. By week five in human gestation, it is expressed throughout the optic vesicle, which then invaginates to form the bi-layered optic cup, where PAX6 is found in both neural and pigmented retinal layers [11]. It is also highly expressed in the anterior segment structures that are derived from the surface ectoderm, including the lens vesicle and corneal epithelium [11,12]. Postnatally, *PAX6* is restricted to retinal ganglion, amacrine and horizontal cells, lens, cornea, conjunctiva, iris, and ciliary body [12,13]. Outside the eye, it is expressed in the pancreas, nasal epithelia, and several distinct regions of the central nervous system (CNS), like the forebrain, hindbrain, and spinal cord [12].

Defects in *PAX6* gene can affect eye development and result in a broad range of clinical phenotypes, with the most common being aniridia, a pan ocular disorder that is primarily characterised by the absence or hypoplasia of the iris, nystagmus, and foveal hypoplasia, accompanied by cataracts, glaucoma and corneal keratopathy [14]. Other ocular phenotypes include microphthalmia, optic nerve anomalies, or anterior segment dysgenesis [15]. Systemic features can include neurodevelopmental abnormalities, like autism and attention deficit hyperactivity (ADHD) disorders, language impairment [16,17,18], and in some cases the absence or malformations of the pineal and pituitary gland [19,20,21,22]. Defects in *PAX6* have also been associated with obesity and diabetes mellitus due to its role in pancreas development [23,24].

The majority of *PAX6* mutations are heterozygous and they result in loss of one allele causing *PAX6* haploinsufficiency. This review will highlight recent advances in *PAX6* function and regulation and will particularly focus on the spectrum of mutations in the eye and resultant genotype-phenotype correlations. 

## 2. *PAX6*

**Figure 1:**
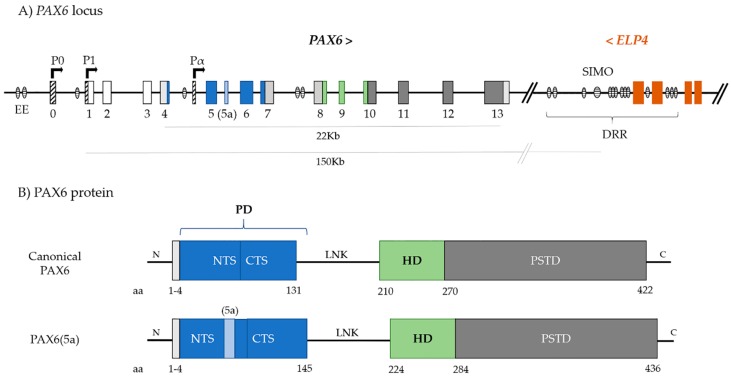
Human *PAX6* locus. (**A**) *PAX6* gene structure in 11p13, with 14 exons (boxes, colours represent respective protein domains), promoters P0, P1 and Pα (boxes with diagonal lines) and regulatory elements (oval shapes with horizontal lines) including ectodermal enhancer (EE), located upstream *PAX6*, and Downstream Regulatory Region (DRR) and SIMO, within the introns of neighbour gene *ELP4* (last four exons represented by orange boxes). EE: ectodermal enhancer; DRR: downstream regulatory region. (**B**) Main PAX6 isoforms, canonical PAX6 and PAX6(5a) and their respective structure with functional domains. PD: paired domain; NTS: N-terminal subdomain; CTS: C-terminal subdomain; LNK: linker region; HD: homeodomain; PST: proline-serine-threonine domain; aa: amino acids.

*PAX6* (OMIM 607108) locus was mapped to the chromosome region 11p13, being 22 Kb in size, but with regulatory regions spanning ~450 Kb of genomic DNA [25,26]. The gene contains 14 exons, with the first three being non-coding (Figure 1A) [27].

*PAX6* encodes a protein whose canonical form is approximately 46 KDa and is formed by 422 amino acids. It has two DNA-binding domains, the paired domain (PD) and the homeodomain (HD), -connected by a linker region (Figure 1B). At the C-terminal, immediately downstream of the HD, there is also a proline-serine-threonine-rich transactivation domain (PSTD), which is required for initiating transcription and it modulates DNA binding by the HD [28,29]. The PD is, in turn, comprised of a N-terminal subdomain (NTS or PAI) and a C-terminal subdomain (CTS or RED) (Figure 1B) [30]. Both subdomains bind the respective consensus DNA sequences and the two major PAX6 isoforms, canonical PAX6 and PAX6(5a), modulate their activity [31,32]. 

PAX6(5a) is formed by the alternative splicing of exon 5a between exons 5 and 6, which results in a slightly larger isoform with 436 amino acids (48 KDa) (Figure 1B). The extra 14 amino acids encoded by exon 5a are inserted in the NTS of the paired domain, blocking its DNA-binding activity, and unmasking DNA-binding activity of the CTS [31,33]. 

A third isoform has been reported in quail and mice—Pax6ΔPD—which lacks the complete PD and it is predicted to result in a truncated protein [34,35]. However, Kim and Lauderdale showed that Pax6ΔPD has a distinct function in mammalian eye development than Pax6 and Pax6(5a), since the overexpression of *Pax6ΔPD*, while using BAC and YAC transgene systems, lead to severe microphthalmia in both wildtype and Pax6-deficient mice [36,37]. Dual reporter systems during mouse embryonic development showed that Pax6ΔPD isoform, although at lower levels than PD-containing isoforms, was mostly detected in the peripheral neural retina and developing ciliary body, but it was absent from developing lens and cornea [36,38].

The regulation and target genes of each isoform in the eye are not entirely understood. It is hypothesized that canonical PAX6 is more prominent during embryonic development of ocular tissues and is related to differentiation and cell fate determination, while PAX6(5a) seems to be more relevant in the later development or postnatally and for cell proliferation [39,40,41]. Accordingly, expression studies in the mouse lens showed that, during embryonic development, canonical *Pax6* expression is much higher (8:1) when compared to *Pax6(5a).* However, in adult eye tissues (lens, cornea and retina), the ratio changes to 1:1 [42,43]. The same tendency was observed in the retina of chick embryos, where canonical *Pax6* is highly expressed during early stages in the eye primordium and lens placode, whereas *Pax6(5a)* expression gradually increases at later stages, particularly in the cornea and lens [39]. The expression of *Pax6(5a)* actually exceeds canonical *Pax6* expression in the posterior retina of chicks, as birds possess a high density of photoreceptors, comparable to the fovea in primates [39]. These expression studies seem to corroborate human phenotypes, since mutations affecting the CTS, which is the main DNA-binding subdomain of PAX6(5a) isoform, have been associated with isolated foveal hypoplasia (discussed in Section 6.3.1.) [33,44,45]. In the cornea, *PAX6* and *PAX6(5a)* are both expressed in the epithelial layer with a correlation between the expression levels of epithelial-specific keratins and PAX6 isoforms. Overexpression studies showed that canonical PAX6 seems to induce *KRT3*, while PAX6(5a) induced *KRT12* expression via each of their respective PD subdomains [46]. 

Although the PAX6 isoforms seem to have independent roles and downstream targets in eye development, the existence of a positive feedback system between Pax6 and Pax6(5a) in mice was also reported [47,48]. Furthermore, the expression ratio between both of the isoforms seems to be essential for normal eye development, and altered ratios has been associated with eye and brain abnormalities [49,50,51]. 

## 3. *PAX6* Regulation

Due to the high similarity of mice, *Drosophila*, or quail *Pax6* to human *PAX6*, these organisms have been extensively used as model systems to understand its function and complex regulatory network. Promoters P0 and P1 mainly regulate the transcription of *PAX6* and, to a lesser extent, by internal promoter Pα, giving rise to the many transcripts that encode the different isoforms (Figure 1) [52,53,54,55]. In situ hybridization experiments in the developing mouse eye showed that P0 promoter initiates gene expression in the cornea and conjunctival epithelia, lens placode, and retina, whereas the P1 promoter mainly initiates transcription in the lens placode, optic vesicle, and CNS, but only weakly in the cornea and conjunctiva. Pα directs the expression in retinal amacrine cells, ciliary body, and iris [41,52,53,55]. 

However, tissue- and time-specific regulation of *PAX6* is still largely unknown. Furthermore, there is no direct relationship between the promoters and expression of specific transcripts. In mice, P0 and P1 both initiate the expression of *Pax6* and Pax6*(5a)* transcripts, while Pα-derived transcripts seems to be more directly linked to the Pax6ΔPD isoform [34,54]. These results point to additional regulation elements that act together with promoters in the complex regulation and expression patterns of *PAX6*. Conserved *cis* regulatory regions have been identified both upstream and downstream of *PAX6* and they are summarized in Table 1. 

The ectodermal enhancer (EE) is located ~3.5 Kb upstream of the P0 promoter and regulates the expression of *PAX6* during the development of surface ectodermal-derived tissues (lens, cornea, conjunctiva) [48,54,60,64,65]. In mice, Pax6 was found to directly interact with the EE for autoregulation mechanisms, but it can also interact with Sox2 and Sox3, transcription factors that are also involved in early eye and lens development [48,66]. The Pax6-Sox2 complex can also act on the Sox2 enhancer N-3, showing a synergistic mechanism between these two transcription factors in the regulation of crystallin gene expression [67]. 

Approximately 150 Kb downstream of *PAX6*, within introns 7 to 9 of neighbour gene *ELP4*, resides an essential region for *PAX6* regulation—Downstream Regulatory Region (DRR). DRR contains several conserved elements that, if absent, affect *PAX6* expression and normal eye development [25,37,57,58]. Although the role of all the elements has not been uncovered, important retinal and iris enhancers are located within this region, since the deletion of DRR in mice completely abolished Pax6 expression in both tissues, as well as in the ciliary body [37]. The deletion of DRR did not dramatically alter Pax6 expression in the lens; the same was observed when the deletion of EE reduced, but did not abolish, Pax6 expression in the lens [64]. These results show that multiple enhancers in multiple regions regulate tissue-specific transcription [37].

Within the DRR, an 800 bp specific enhancer element—SIMO—was found to have a PAX6 PD consensus binding sequence, guiding expression in early and later stages of eye formation, i.e., early surface ectoderm, lens, and neural retinal differentiation, as well as in adulthood, in the lens epithelium, retina, and iris [26]. SIMO was shown to be critical for *Pax6* expression, as the disruption of this element alone was sufficient for abolishing expression in the developing lens in transgenic mice and zebrafish [26]. Importantly, deletion or point mutations in SIMO were described to cause aniridia phenotype in humans (discussed in Section 4.4.) [26,68]. However, the disruption of *PAX6* expression in the lens when SIMO is affected is most likely due to a self-regulation mechanism of PAX6 itself, which, as in EE, can bind to SIMO via PD and regulate its own expression during eye development in a positive feedback loop [26]. In the mouse lens, two transcription factors from the TALE homeoproteins family, Meis1 and Meis2, have been identified as upstream regulators of Pax6 by regulating the activity of both EE and SIMO enhancers [69,70]. However, the exact role and regulation mechanisms of these regions on *PAX6* expression in other eye tissues are still largely unknown.

**Figure 2:**
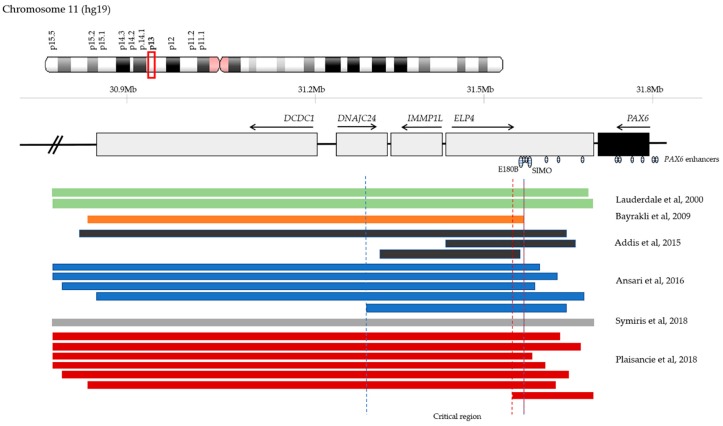
Schematic representation of previously reported deletions in 11p13 encompassing regulatory regions 3’ of *PAX6*. Genes are represented by grey boxes and arrows indicate direction of transcription. *PAX6* is highlighted in black. Known *PAX6* enhancers are indicated by oval shapes with horizontal lines with focus on SIMO and E180B. Coloured bars represent deletions identified in different cohorts of aniridia patients without *PAX6* mutations [68,72,73,75,76,77]. The vertical dashed lines indicate a common region deleted in all mentioned aniridia patients, which was defined first as 245 Kb long by Ansari et al. (blue dashed lines) and later reduced to 18 Kb by Plaisancie et al. (red dashed lines) [72,75]. Genomic coordinates are based on human genome assembly hg19.

Comparative genomic hybridisation (CGH) analysis of aniridia patients that lacked mutations in *PAX6* coding sequence, pointing to additional regulatory elements located in this region, have identified deletions in *PAX6* 3’ regulatory region (Figure 2) [71,72,73,74,75]. As a result, Ansari et al. proposed a critical region for *PAX6* transcriptional activation that spanned approximately 245 Kb and encompassed neighbour genes *DNAJC24*, *IMMP1L*, and *ELP4* [72]. More recently, Plaisancie et al. refined it to a 18 Kb region within *ELP4* that does not include SIMO, but another highly conserved enhancer in the DRR, E180B (Figure 2) [56]. These findings highlight a possible role of E180B in aniridia [75]. 

## 4. *PAX6* Mutation Spectrum

**Figure 3:**
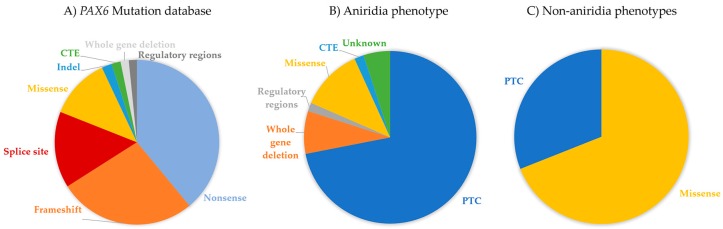
Distribution of different mutation types in the *PAX6* Mutation Database. (**A**) All of the mutations reported in the database. (**B**) Mutation frequencies associated to aniridia. (**C**) *PAX6* mutation distribution in non-aniridia phenotypes. PTC (premature termination codon) include all mutations predicted to cause a premature stop codon and include nonsense, frameshift and splice site variants. CTE refer to variants that cause C-terminal extension of PAX6.

The “*PAX6* Mutation Database” (http://lsdb.hgu.mrc.ac.uk/home.php?select_db=PAX6) catalogs all reported mutations in *PAX6* and had, until its last update in 2018, nearly 500 unique variants registered. Intragenic mutations cover ~96% of variants in the database, while the remaining 4% represent the whole gene deletions or variants described in 5’ and 3’ regulatory regions. 80% of intragenic mutations are spread through the complete coding sequence of the gene, but the majority seem to be located within exon 5, 6 and 9, which represent the paired (5 and 6) and homeo- (9) domains of PAX6 protein. While considering the mutation type, the most common intragenic mutations that were observed in *PAX6* are nonsense (39%), followed by frameshifts (27%), missense (12%), splice site (15%), small indels (2%), and C-terminal extension (or run-ons) (2%) (Figure 3) [15,78].

### 4.1. Premature Termination Codon (PTC) Mutations—Nonsense, Frameshift, and Splice Site Variants

Nucleotide changes that introduce a premature termination codon (PTC) are the most common mutations that are found in *PAX6*. Nonsense, frameshift (out of frame insertions or deletions), and most splice site mutations result in the insertion of a PTC and the consequent termination of translation, accounting for 65–70% of all *PAX6* mutations in the database (Figure 3A). Patients with PTC variants tend to present classical aniridia phenotype [15]. 

Although PTC mutations were first thought to cause truncated proteins with a dominant negative effect, it is now accepted that mRNA transcripts containing PTCs located until 50 bp upstream of the last exon junction may be subjected to nonsense-mediated decay (NMD) [79]. Hence, PTCs in *PAX6* mostly result in the degradation of the mutated transcript and the consequent loss of 50% of PAX6 protein levels [15,80]. Four specific nonsense variants are the most common PTC mutations and they account for more than 20% of all entries in the database: c.607C>T, p.Arg203* (exon 8); c.718C>T, p.Arg240* (exon 9); c.781C>T, p.Arg261* (exon 10); and, c.949C>T, p.Arg317* (exon 11) [15]. These mutations are located in known methylated CpG islands in exons 8–13, which constitute mutational “hotspots” for aniridia [78]. 

Further evidence that NMD is acting on PTC-containing *PAX6* transcripts is the absence of these variants at the 3’ end of the gene [78]. PTCs in this region are expected to escape NMD and they would result in truncated proteins with a possible dominant negative effect and more severe phenotypes [81,82]. However, within the region that was predicted to escape NMD, i.e., the last 50 bp from exon 12 and exon 13, there are no nonsense mutations reported aside from c.1183G>T (p.Gly395*) in the last nucleotide of exon 12, but with predicted mRNA missplicing [83], and all frameshift changes are predicted to cause C-terminal extensions [78].

### 4.2. C-Terminal Exptension (CTE) Variants 

Frameshift or point mutations in *PAX6* that alter the stop codon location and allow for translation to continue into the 3’UTR region are less frequent than the variants described earlier, with only 13 entries being reported. Large aniridia cohort studies show that CTEs are associated with aniridia-like phenotypes, with a severity comparable to PTC-causing variants [84,85,86]. These observations seem to suggest that CTE mutations also generate *PAX6* haploinsufficiency; however, NMD does not degrade these transcripts, since there is no PTC introduced, which means that these changes should indeed produce a longer protein that would most likely be unstable with the affected PST domain transactivation activity. The mechanisms of how CTE mutations cause haploinsufficiency are still unexplained.

### 4.3. Missense Variants

Missense mutations, which cause one amino acid to be replaced by a different one during translation, are reported in ~12% of all entries in the *PAX6* Mutation Database. They are more concentrated in the PD and functional studies predict these variants cause differences in DNA binding and the transactivation activities of PAX6 [87,88,89,90]. These mutations are usually associated with milder, but atypical ocular phenotypes, in some cases without the presence of iris defects [86]. In fact, Tzoulaki et al. reported that missense mutations in *PAX6* are responsible for nearly 70% of non-aniridia eye disorders that were registered in the database (Figure 3C) [78], like microphthalmia, optic nerve anomalies, coloboma, isolated foveal hypoplasia, and anterior segment dysgenesis (discussed in Section 6.3.) [44,90,91,92,93].

### 4.4. Non-Coding Variants 

An increasing number of reports point to the implication of mutations in non-coding regions in neurodevelopmental and eye disorders [94,95,96]. In the *PAX6* Mutations Database, approximately 15% of all variants are located in the intronic regions and they are generally associated with classical aniridia phenotypes. Although the great majority are located in donor and acceptor sites at the intron-exon borders, deep intronic variants have recently been found in large aniridia patient cohorts [75,83]. The deletions or point mutations in non-translated 5’ and 3’UTR of *PAX6* have also been reported [75,97,98]. Minigene assays or *in silico* analysis revealed that most of these variants are likely to affect the normal splicing patterns, which results in the formation of PTCs and the consequent *PAX6* haploinsufficiency [99].

Changes in 3’ regulatory *PAX6* regions were also identified in patients presenting with classical aniridia. A single nucleotide change (chr11: 31,685,945G>T) in the SIMO enhancer, ~150Kb downstream of *PAX6*, was described by Bhatia et al., who showed that the phenotype is due to the change affecting a PAX6 recognition site, disrupting its autoregulation loop, and ultimately resulting in decreased *PAX6* expression and haploinsufficiency [26]. Several deletions that encompass other 3’ regulatory regions have also been reported to cause aniridia (Figure 2) [71,72,73,74,75,76,77]. 

### 4.5. Chromosomal Rearrangements and Large Deletions 

Chromosomal rearrangements (deletions, duplications, translocations, or inversions) involving part or whole *PAX6* gene or regulatory elements account for up to 10% of all aniridia cases. Some reports suggest that the *PAX6* deletions are more frequent in sporadic as compared to familial aniridia patients, while others found no significant difference [84,100,101,102]. Large deletions that encompass *PAX6* and other neighbour genes, such as *WT1*, result in systemic disease, such as WAGR, caracterised by the presence of Wilms tumour, aniridia, genitourinary anomalies, and retardation characterize (described in Section 6.2). 

### 4.6. Biallelic Mutations 

Mutations that affect both *PAX6* alleles have rarely been described and cause very severe ocular and neurodevelopmental abnormalities, in most cases leading to embryonic death [103,104]. A surviving patient with compound heterozygous mutations in *PAX6* was described as having microphthalmia, neonatal diabetes mellitus, hypopituitarism, and microcephaly, as well as trisomy 21. The patient inherited a missense mutation affecting the PD from the father (c.112C>T, p.Arg38Trp), who had microcornea and severe cataracts, and a nonsense mutation in HD from the mother (c.718C>T, p.Arg240*), who presented with classical familial aniridia [105]. It is plausible to assume that, although affected, the missense variant still contributed for some amount of PAX6 function, since *PAX6* is a very dosage-sensitive gene. Another patient with compound heterozygous nonsense mutations (c.607C>T, p.Arg203* and c.1058C>G, p.Ser353*), presenting with anophthalmia, severe CNS defects, including microcephaly and the complete absence of the corpus callosum and olfactory bulbs, sadly died a few days after birth [103]. 

## 5. Inheritance of *PAX6* Mutations

Most *PAX6* mutations causing aniridia are heterozygous, sporadic, or familial in an autosomal dominant manner, with significant phenotypic variability. It is estimated that nearly two-thirds of aniridia cases are familial, while the remaining third are considered to be sporadic [86,106]. Accordingly, sporadic occurrence was described in nearly 40% of entries in the *PAX6* Mutation Database. Up to one-third of sporadic cases of aniridia are associated with *PAX6* and *WT1* deletions, while the remaining two-thirds are considered to be most likely caused by *de novo* point mutations [89]. 

Recent studies reported that the rate of mosaicism could be as high as 17.5% among apparent *de novo* cases for different dominant disorders [83,107]. Indeed, several reports have suggested mosaicism as the cause for the variable phenotypes seen in some sporadic *PAX6*-affected patients [83,93,108,109]. A recent study from Tarilonte et al. proved the existence of post-zygotic parental mosaicism in three unrelated Spanish families with variable aniridia or microphthalmia phenotypes caused by heterozygous nonsense (c.771G>A, p.Trp257* and c.120C>A, p.Cys40*) or missense (c.178T>C, p.Tyr60His) *PAX6* mutations, respectively [110]. Quantitative analysis of parental *PAX6* gene showed that all of the proband’s fathers have mutant allele fractions that range from 13 to 29%, depending on the tissue analysed, and have mild or no ocular features as compared to their affected offspring. Similarly, Bai et al. reported the presence of male gonadal mosaicism in a Chinese family with aniridia caused by PTC-inducing mutation c.879_880delCA, p.Ser294Cysfs*46 [109]. 

The presence of parental mosaicism in *PAX6* contributes to partially explaining intra-familiar variabilities very often seen in patients. Furthermore, mosaicism is particularly relevant in sporadic aniridia cases, as it might be the underlying transmittance mechanism and not, in fact, *de novo* mutations, which has deep implications for genetic testing and counselling.

## 6. Genotype-Phenotype Correlations

### 6.1. Aniridia (MIM 106210)

Aniridia is a pan-ocular disorder that bilaterally affects the formation of the iris, cornea, lens, fovea, and optic nerve. Its prevalence is 1:40,000–100,000 with complete penetrance and variable expressivity [15,100,111]. 

**Figure 4:**
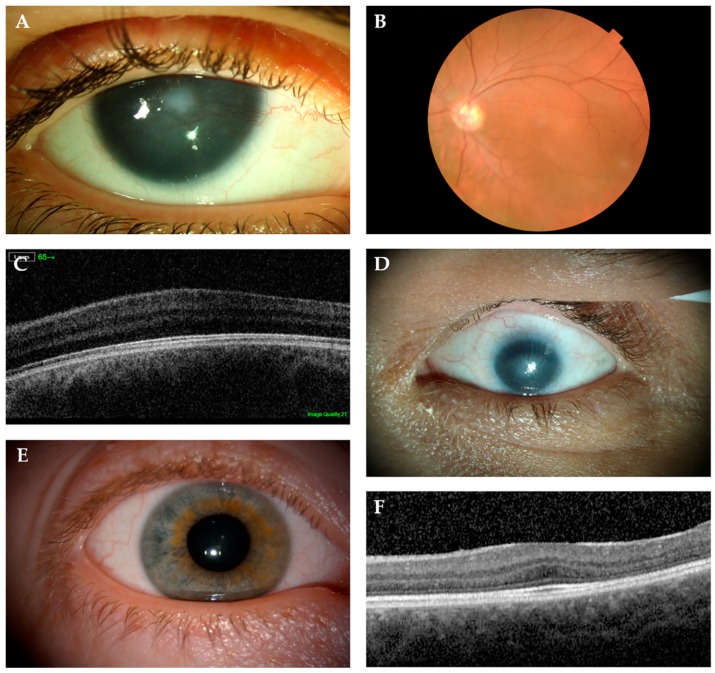
Phenotypic spectrum of patients with *PAX6* mutations. (**A**) Classical aniridia phenotype showing complete iris hypoplasia with central corneal opacity and vascularisation, (**B**) fundus imaging indicating absence of foveal reflex and (**C**) optical coherence tomography (OCT) of the macula showing foveal hypoplasia. (**D**) Left eye of a patient diagnosed with bilateral microphthalmia with microcornea. (**E**) Patient diagnosed with dominant nystagmus with no iris abnormalities and (**F**) foveal hypoplasia on OCT.

The most obvious ocular feature is complete or partial iris hypoplasia, being accompanied by nystagmus and foveal hypoplasia, with the latter being the main cause of reduced visual acuity from birth. Further vision loss can occur from later onset cataracts, aniridia-related corneal keratopathy (ARK), and glaucoma (Figure 4A–C). Cataracts tend to develop during the late teens to early adulthood in 50–85% of aniridia patients, while up to two-thirds of patients can develop glaucoma between late childhood and early adulthood [15,112]. ARK is the most relevant feature contributing to visual loss in aniridia patients and it affects ~20% patients, but up to 90% can present with corneal irregularities [113]. Foveal hypoplasia and nystagmus were estimated to range between 85–95% of aniridia patients [86]. Optic nerve hypoplasia, although less common, is associated with ~10% of aniridia cases [90,114]. 

However, aniridia patients can present with a wide phenotypic spectrum without clear correlation between genotype and phenotype, even in patients within the same family [15]. Accordingly, a recent report from Pedersen et al. showed that family members with the same *PAX6* mutation, a 2 bp deletion in intron 2, presented with variable iris involvement, which ranged from almost normal to no iris, as well as different degrees of foveal hypoplasia [115,116]. In contrast, Lagali et al. recently found a correlation between *PAX6* variants and the severity and progression of ARK [117,118]. In a cohort of 46 aniridia patients, the authors found a minimal level of keratopathy and an increase in cornea thickness in all aniridia patients from early age. Patients with whole gene deletions presented the most severe and early onset ARK, followed by those with PTC or CTE mutations. Patients with missense mutations showed milder non-progressive ARK and, lastly, non-*PAX6* mutations had the mildest forms of disease and generally the best visual acuity [118]. However, the clinical phenotype of ARK is heterogenous and patients with the same mutation can often display different degrees of ARK. 

*PAX6* haploinsufficiency, which is caused by intragenic PTC-causing mutations, whole gene deletions, or inactivation of regulatory regions, is the cause of up to 85% of aniridia cases (Figure 3B). Independent of the type or location of the PTC-containing transcripts, NMD is thought to be activated, resulting in null alleles. Iris hypoplasia is the most common feature in patients with mutations that cause *PAX6* haploinsufficiency, whereas patients with missense mutations tend to have less affected iris [86]. CTE variants usually translate to severe iris hypoplasia similar to PTCs, although Hingorani et al. examination of 10 patients with CTE showed that the iris phenotypes tend to be milder in these patients compared to patients with PTCs in the same study [86,119]. 

Aniridia-like phenotypes have been found in patients with rare variants in *FOXC1* and *PITX2* [72,120,121]. These genes are usually associated with anterior segment dysgenesis (ASD), which are characterized by iris hypoplasia (or atrophy) and corectopia, and, frequently, childhood-onset glaucoma. However, patients with ASD usually have better visual acuity than aniridia patients, without nystagmus or foveal abnormalities [15,122]. In mice, *Foxc1* was recently found to be a downstream target of Pax6 in the iris, and the deletion of this gene also caused cornea neo-vascularisation, which indicated that both genes belong to a common network in the formation of the anterior segment [123,124]. Recently, missense mutations in *TRIM44* gene, located 4 Mb away from *PAX6*, were identified in a Chinese family with aniridia, cataracts and glaucoma [125]. TRIM44 was shown to negatively regulate *PAX6* expression, but only one pedigree was reported to date. 

It is estimated that 5% of aniridia patients remain without molecular diagnosis, pointing to more *PAX6* regulatory regions, modifiers, or even novel genes to be discovered [102]. 

### 6.2. WAGR (MIM 194072)

WAGR (Wilms tumour, Aniridia, Genitourinary abnormalities and mental Retardation) is an autosomal dominant disorder with a prevalence of 1:500,000 [15]. Chromosomal rearrangements or small deletions in 11p region encompassing *PAX6* and *WT1* loci cause it, but can be variable in size [71,126]. Approximately 30% of patients with sporadic aniridia suffer from this syndrome and diagnosed children have 50 to 70% risk of developing Wilms tumour involving the kidney, so regular screening is necessary for increasing the detection and obtaining better prognosis [127]. 

While the absence of one copy of *PAX6* and *WT1* is established as the cause for aniridia and Wilms tumour (and genitourinary anomalies), respectively, the genetic causes that are behind the neurodevelopmental defects are less clear. Within the critical region in 11p, several genes have been associated with neurodevelopmental problems, like autism and ADHD. *BDNF*, a gene encoding the brain-derived neurotrophic factor, is located ~4 Mb from *PAX6* in 11p14.1. Patients with *BDNF* haploinsufficiency have variable degrees of developmental delay, as well as behavioural problems [128,129]. *SLC1A2*, encoding a glutamate transporter, and *PRRG4*, encoding a vitamin K-dependent membrane protein, are also located in 11p13-p12, a region that is identified by linkage analysis as an autism candidate region [130]. However, *PAX6* itself should also be considered, since some patients with *PAX6* intragenic mutations present with cerebral abnormalities as well as development delays and autism [16,17,131]. 

WAGRO (MIM 612469) is a variant syndrome of WAGR that includes obesity [132,133]. Patients that were diagnosed with WAGRO have deletions that also encompass *BDNF*. *BDNF* is also involved in energy homeostasis in humans and haploinsufficiency is correlated with higher BMI (Body Mass Index), increased appetite, and childhood-onset obesity compared to WAGR patients without *BDNF* deletion [126,128].

### 6.3. Non-Aniridia Phenotypes

#### 6.3.1. Isolated Foveal Hypoplasia

**Figure 5:**
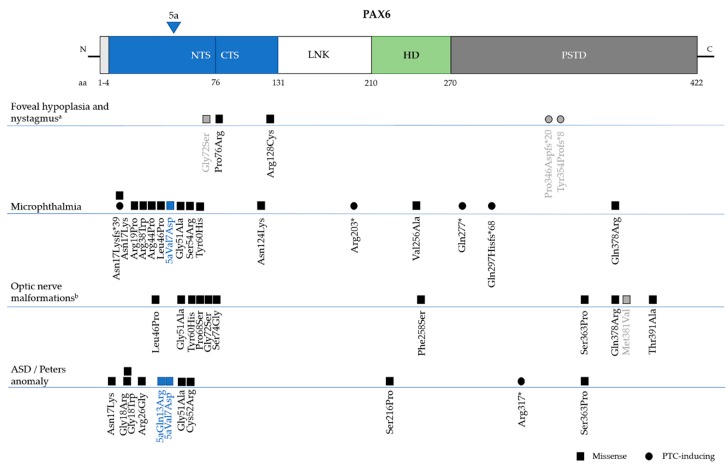
Spectrum of coding *PAX6* mutations causing non-aniridia phenotypes. A representation of canonical PAX6 structure is shown with respective domains and amino acid distribution. Mutations and respective phenotypes were collected from the *PAX6* Mutation Database and the Human Genome Mutation Database (HGMD). The majority of mutations are included in the paired domain (NTS and CTS) and constitute missense mutations (squares). Mutations in alternative exon 5a are shown in blue. ^a^ Isolated foveal hypoplasia and nystagmus without iris abnormalities; variants shown in grey show cases presenting full iris but with possible mild structural defects [86]; ^b^ Optic nerve malformations include optic nerve coloboma, aplasia, and morning glory disc; NTS: N-terminal subdomain; CTS: C-terminal subdomain; LNK: linker region; HD: homeodomain; PSTD: proline-serine-threonine domain; aa: amino acids; ASD: anterior segment dysgenesis.

Isolated foveal hypoplasia, which is usually accompanied by nystagmus, has been described in few families with *PAX6* missense mutations in the paired domain: c.227C>G, p.Pro76Arg and c.382C>T, p.Arg128Cys [44,45,134]. These mutations are located in the CTS subdomain of PD, which modulates the DNA-binding activity of PAX6(5a) isoform [32]. It was previously reported that the PAX6(5a) isoform was highly expressed in the fovea, and that mutations in exon 5a, which affect CTS binding activity, also caused foveal hypoplasia (not isolated) [39]. These results point to foveal hypoplasia being linked to variants that affect the PAX6(5a) isoform in particular, but further experimental evidence is required to confirm this. However, it should also be noted that a missense (c.214G>A, p.Gly72Ser) in the end of NTS and two PTC mutations in the PSTD (c.1035_1048del14, p.Pro346Aspfs*20 and c.1061_1070del10, p.Tyr354Cysfs*8) were also identified by Hingorani et al. in patients with foveal hypoplasia and mild structural abnormalities in the iris (Figure 5) [86]. 

#### 6.3.2. Microphthalmia, Anophthalmia and Coloboma (MAC)

MAC is a group of developmental eye disorders characterised by reduced size orabsence of the ocular globe and it is caused by mutations in more than 90 genes, including *PAX6* (Figure 4) [92]. Of the 13 different mutations that are associated with microphthalmia in the *PAX6* Mutation Database, the vast majority (8) are missense, although PTC-inducing mutations have been reported (two nonsense and two frameshifts) (Figure 5) [108,110,135,136]. Bilateral microphthalmia in a patient with WAGR syndrome was also identified [137].

MAC phenotypes are significantly associated with mutations that are likely to disrupt the PAX6-DNA interaction, with mutations being more common in the PD, which is the domain that is known to interact with SOX2 [67,138,139,140]. Mutations in *SOX2* are the most common cause of bilateral anophthalmia and severe microphthalmia [89,90]. Accordingly, a recent report has linked novel missense mutations in *PAX6* (c.160A>C, p.Ser54Arg and c.372C>A, p.Asn124Lys) to severe bilateral microphthalmia, with phenotype resembling *SOX2*-associated MAC [140]. The authors showed that these mutations significantly reduce the binding affinity of PAX6 to a specific regulatory sequence in the EE, LE9, which also synergistically responds to SOX2 [48]. This could lead to an inability of both PAX6 and SOX2 to cooperatively bind to LE9 or other target DNA sequences, which suggests that, aside from being required during lens development, the PAX6-SOX2 partnership can have an additional role earlier in eye field development.

Anophthalmia is associated with homozygous *PAX6* variants, where there is biallelic loss of function. The affected individuals are usually still births or die soon after birth with severe brain abnormalities [27,104]. 

The most common form of ocular coloboma resulting from *PAX6* defects is that affecting the iris, which have been identified in patients with both nonsense, missense, and frameshift changes in *PAX6*, as well as the deletion of 3’regulatory region [141,142,143,144]. Optic nerve coloboma are generally associated with microphthalmia, with missense mutations affecting all of the functional domains of PAX6 (Figure 5) [91,92]. It is thought that the inability of PAX6 to bind *PAX2* promoter and repress its expression in the optic cup during early eye development cause *PAX6*-related optic nerve anomalies. PAX2 is another PAX-family transcription factor that is mainly expressed in the optic stalk and represses *PAX6* expression in this region. This mutual repression is essential for defining the boundaries between the optic stalk, which will later form the optic nerve, and the optic cup [90,145].

#### 6.3.3. Gillespie Syndrome (MIM 206700)

Another syndrome with aniridia-like features is Gillespie syndrome (GS), which is a rare disorder that is characterized by nonprogressive cerebellar ataxia, intellectual disability, hypotonia, and iris hypoplasia with the presence of scalloped edges, fixed dilated pupils, and remnants of pupillary membrane [102]. Mutations in the *ITPR1* gene were identified as causative in GS [146,147]. However, a T>A substitution in intron 2 of PAX6 was identified in two individuals that were described with Gillespie syndrome, but with atypical features, like corectopia and ptosis [148]. Later, a chromosomal deletion encompassing the 3’ regulatory region of PAX6 was also identified in a patient that was diagnosed with Gillespie syndrome [72].

#### 6.3.4. Anterior Segment Dysgenesis–Peters Anomaly (MIM 604229)

Peters anomaly is part of a larger spectrum of anterior segment dysgeneses characterized by abnormalities in the cornea, iris and lens, including central cornea opacity, defects in corneal stroma and Descemet’s membrane, and iridocorneal and corneolenticular adhesions [122]. Mutations in *PAX6*, but also *PITX2* and *CYP1B1*, have been identified in patients with Peters anomaly [122,149]. In the *PAX6* Mutation Database there are 11 different mutations associated with this syndrome: 10 are missense mutations, localized primarily in the PD, and one is a nonsense variant in the PSTD (Figure 5) [33,45,149,150]. 

## 7. Conclusions

PAX6 plays a number of important roles both during eye development and maintenance of adult eye tissues. Defects in PAX6 lead to the perturbations in the dosage, time, and tissue specific expression, as well as disruption of its regulatory network. Due to this complex picture, individuals with mutations affecting *PAX6* present highly variable pan-ocular features, which makes genotype-phenotype correlations difficult to establish.

Developments in transcriptomics and epigenomics have allowed for increasing the understanding of PAX6 transcriptional targets, regulators, and interactors in the eye [12,151,152,153]. Accordingly, growing evidence supports the importance of *PAX6* regulation through microRNAs, as well as long non-coding RNAs [154,155,156,157,158,159]. These key advances mainly arose from mouse and *Drosophila* studies. However, it is still far from clear how PAX6 operates during eye development and how mutations translate into the variability of phenotypes that are described in this review. More representative models are needed for this purpose, and to overcome innate differences in eye development between human and other model organisms.

The generation of induced pluripotent stem cells (iPSCs)s allowed for the development of more representative in vitro models of human eye diseases, as well as of human eye development. This strategy would be particularly suitable for complex diseases, like aniridia, since iPSCs have the ability to differentiate into different cell types. Several ocular cell types where PAX6 activity is important have been successfully derived from iPSCs, like corneal and lens epithelial cells [157,160,161,162]. Additionally, iPSC-derived retinal organoids are now standardly used to study disease mechanisms and develop novel therapeutic approaches for eye disorders, like inherited retinal dystrophies [163,164,165,166,167]. However, this promising approach has not yet been applied to study *PAX6*- related eye diseases.

The majority of *PAX6* mutations result in null alleles and consequent *PAX6* haploinsufficiency and they are known to cause aniridia. Hence, understanding the *PAX6* dosage requirements in each affected tissue is of particular importance, not only to understand phenotypic outcomes, but also to predict therapeutic targets. Nonsense suppression therapy targets PTCs that are caused by nonsense mutations and bypasses them to produce full length protein [168]. A clinical trial on the oral administration of ataluren (PTC124) in aniridia patients is currently ongoing (NCT02647359), after promising results were seen in Sey^+/−^ mice that were treated with this compound [169,170]. Postnatal administration of ataluren was shown to increase the levels of Pax6 protein up to 80% of control levels, improving lens and corneal epithelium morphology, as well as retina function, as shown by increased ERG responses. However, the exact mechanism of action of ataluren is still unknown, and there is criticism over the mode of drug administration, as topical/local application might permit lower dose, higher penetration, and less off-target effects. The results from the clinical trial are eagerly awaited.

## Figures and Tables

**Table 1:** Summary of conserved regulatory elements in the *PAX6* locus and their regulation of *PAX6* ocular and extra-ocular expression.

Regulatory Element	Distance to *PAX6* (Kb)	Location	Eye Expression	Extra-Ocular Expression	Reference
RB	−215	Intron *ELP4*		Diencephalon, telencephalon, pineal gland	[37]
E180B	−176	Intron *ELP4*	RGCs and optic nerve	Trigeminal ganglia, dorsal spinal cord neurons	[56]
HS8B	−177	Intron *ELP4*			[25]
HS8A	−176	Intron *ELP4*			[25]
HS6	−172	Intron *ELP4*	Neural retina	Neural structures	[57]
HS5	−168	Intron *ELP4*	Optic cup and neural retina	Diencephalon	[57]
HS3	−162	Intron *ELP4*	Neural retina		[25]
HS2	−161	Intron *ELP4*	Neural retina		[25]
SIMO	−153	Intron *ELP4*	Lens and neural retina	Diencephalon, hindbrain, neural tube	[25]
E120	−126	Intron *ELP4*		Olfactory bulbs, brain	[56]
E100	−104	Intron *ELP4*	Neural retina	Diencephalon, olfactory region	[58]
E60A	−54	Intron *ELP4*	Optic cup and neural retina	Neural structures	[57]
7CE1	−18	Intron *PAX6*	Late eye development		[59]
NRE	−13	Intron *PAX6*	Neural retina, ciliary body, iris		[54]
0CE1	−1	5’UTR *PAX6*			[26]
agCNE14 (P2)	4			Pancreas	[56]
EE	5		Cornea, lens, conjunctiva and lacrimal gland		[54,60]
agCNE12 (P)	7			Pancreatic islets	
agCNE11	8			Pineal gland	[56]
agCNE10 (Up-9)	9			No specific pattern	[56]
agCNE9 (Up-10)	12			Pineal gland	[56]
Mouse-like Paupar	54				[61]
PE3 (E-52)	57			Developing pancreas and brain	[62]
E-55/C	59			No specific pattern	[56]
E-55/B	60			No specific pattern	[56]
E-55/A	70			Hindbrain	[56]
E-72	71			No specific pattern	[56]
PE4 (E120)	110			Developing pancreas, olfactory tract, cerebellum, hindbrain	[62]
agCNE5 (Id855)	178			Forebrain	[56]
agCNE4	186			Hindbrain	[56]
agCNE3	214			Olfactory bulbs	[56]
E200	214			Olfactory bulbs, cerebellum	[63]
agCNE1	224			Trigeminal ganglia, dorsal spinal cord neurons	[56]
E250	247			No specific pattern	[56]
